# Platelet factor 4 polyanion immune complexes: heparin induced thrombocytopenia and vaccine-induced immune thrombotic thrombocytopenia

**DOI:** 10.1186/s12959-021-00318-2

**Published:** 2021-09-15

**Authors:** Payel Datta, Fuming Zhang, Jonathan S. Dordick, Robert J. Linhardt

**Affiliations:** Heparin Applied Research Center, Center for Biotechnology and Interdisciplinary Studies, Rensselaer Polytechnic Institute, Troy, NY 12180 USA

**Keywords:** Biosynthetic heparins, HIT, VITT, COVID-19, PF4, Platelet factor 4/polyanion complex

## Abstract

**Background:**

This is a review article on heparin-induced thrombocytopenia, an adverse effect of heparin therapy, and vaccine-induced immune thrombotic thrombocytopenia, occurring in some patients administered certain coronavirus vaccines.

**Main body/text:**

Immune-mediated thrombocytopenia occurs when specific antibodies bind to platelet factor 4 /heparin complexes. Platelet factor 4 is a naturally occurring chemokine, and under certain conditions, may complex with negatively charged molecules and polyanions, including heparin. The antibody-platelet factor 4/heparin complex may lead to platelet activation, accompanied by other cascading reactions, resulting in cerebral sinus thrombosis, deep vein thrombosis, lower limb arterial thrombosis, myocardial infarction, pulmonary embolism, skin necrosis, and thrombotic stroke. If untreated, heparin-induced thrombocytopenia can be life threatening. In parallel, rare incidents of spontaneous vaccine-induced immune thrombotic thrombocytopenia can also occur in some patients administered certain coronavirus vaccines. The role of platelet factor 4 in vaccine-induced thrombosis with thrombocytopenia syndrome further reinforces the importance the platelet factor 4/polyanion immune complexes and the complications that this might pose to susceptible individuals. These findings demonstrate, how auxiliary factors can complicate heparin therapy and drug development. An increasing interest in biomanufacturing heparins from non-animal sources has driven a growing interest in understanding the biology of immune-mediated heparin-induced thrombocytopenia, and therefore, the development of safe and effective biosynthetic heparins.

**Short conclusion:**

In conclusion, these findings further reinforce the importance of the binding of platelet factor 4 with known and unknown polyanions, and the complications that these might pose to susceptible patients. In parallel, these findings also demonstrate how auxiliary factors can complicate the heparin drug development.

## Introduction

Heparin is an essential life-saving drug and is extensively used as an anticoagulant [1]. Approximately 175 metric tons of heparin are used annually worldwide. Pharmaceutical heparin is animal-sourced and extracted from the porcine intestinal mucosa [2, 3]. Concerns over animal-sourced biopharmaceuticals, including contamination with prions, viruses, or processing impurities, has led to efforts to develop bioequivalent heparins from non-animal sources [4–22], which may be safer compared to animal-sourced products. Furthermore, bioequivalent heparins may diversify the existing supply chain [2].

Heparin-induced thrombocytopenia (HIT) is an adverse effect of heparin therapy. Type 1 HIT is a nonimmune disorder that results from the direct effect of heparin on platelet activation. Type 2 HIT is an immune-mediated disorder that typically occurs 4–10 days after exposure to heparin [23]. HIT occurs in a small fraction of patients (0.2 to 3%) undergoing heparin therapy [24]. HIT is a complicated pathology and associated with life-threatening thromboembolic complications [23]. Prompt and accurate diagnosis is critical for HIT treatment. Therefore, understanding the biology of HIT is imperative towards HIT diagnosis and the development of bioequivalent heparins.

The current review includes an overview of: (1) heparin; (2) the biology of HIT; (3) current clinicopathological diagnosis of HIT; and (4) the regulatory importance on the development of bioequivalent heparins.

## Heparin

Heparin is a negatively charged linear mucopolysaccharide and is naturally found in the granules of mast cells [25]. Heparin is composed of linear chains of disaccharide units of D-glucosamine and uronic acid (L-iduronic acid or D-glucuronic acid) [25]. These sugar moieties are sulfated (2.0–2.5 sulfate groups per disaccharide unit) The sulfation patterns on heparin dictate the interaction of heparin with various ligands [25], including proteins, which is domain-specific or charge-dependent [25–31]. Anticoagulant heparin has characteristic sulfated pentasaccharide domains that bind to antithrombin III (ATIII), which leads to inhibition of the blood coagulation cascade and results in anticoagulation [25, 32]. Heparin may interact with a myriad of ligands in a charge-dependent manner [25, 33, 34]. For example, the highly negatively charged heparin interacts with the positively charged proteins, such as platelet factor 4 (PF4, CXCL4) [33]. Importantly, the interaction of heparin and PF4 is associated with immune-mediated HIT [33].

Since 1977, heparin has been listed as essential medicine by the World Health Organization [35]. The therapeutic effects of heparin are based on the fact that heparin binds to specific proteins in the blood coagulation pathway and initiates a reaction cascade that leads to blood anticoagulation. As part of this cascade, heparin binds to ATIII, causes a conformational change in the protein, which increases the affinity of ATIII to bind to and inactivate coagulation enzymes, specifically, thrombin (Factor IIa) and Factor Xa. Heparin is used for the prophylaxis and treatment of medical conditions and surgical procedures that can lead to blood clotting, including acute myocardial infarction, arterial and venous thromboembolism, and lung thromboembolism. Different therapeutic heparins include unfractionated heparin (UFH, MW: 16 kDa), low-molecular weight heparin (LMWH, MW: 4.5 kDa), and ultra-low-molecular weight heparin, fondaparinux (ULMWH, MW: 1728) (Table 1). Current drug development efforts have focused on generating therapeutic heparins from non-animal sources and using chemoenzymatic methods.

**Table 1:** Comparison of UFH, LMWHs and fondaparinux

Anticoagulation therapy	UFH	LMWH	Fondaparinux
**Nomenclature**	Unfractionated heparin	Two LMWHs based formulation (1) dalteparin (Fragmin®) and (2) enoxaparin (Lovenox®)	Fondaparinux (Arixtra®)
**Mechanism of action**	Bind to AT and increases the affinity of AT to bind and inactivate both Fxa and thrombin	Bind to AT and increases the affinity of AT to bind and inactivate Fxa. The effect on inactivation of thrombin is relatively less.	Bind to AT and increases the affinity of AT to bind and inactivate Fxa.
**Half life**	60–120 min	(4–7 h) Enoxaparin 2–5 (dalteparin)	17–21 h
**Neutralization with protamine sulfate**	Yes	Yes	-NA-
**Clearance**	Hepatic & Reticulo-endothelial system No renal adjustments	Renal	Renal
**Ability to cause HIT**	Yes	Yes	Low; Cases reported

### Heparin derivatives with reduced HIT

Currently, heparins are manufactured by purification from animal tissues such as porcine intestines and bovine lungs [2, 3]. Due to the poor control of animal tissues, potentially limited availability, impurities, viruses, prions, and contamination, there has been increased interest in novel approaches for heparin production [36]. These novel approaches include chemical synthesis, chemoenzymatic synthesis, bioengineered/biosynthetic heparin, and recombinant heparin by metabolic engineering. The heparin derivative products, such as ULMWHs and LMWHs, can result in reduced HIT.

### Low molecular weight heparins (LMWHs)

LMWHs are manufactured by the controlled chemical or enzymatic depolymerization of unfractionated heparin. The interaction between heparin and PF4 is size-dependent [37]. As a result, the absolute risk of HIT is 2–3% for unfractionated heparin, but LMWH can reduce the risk of HIT to 0.2–0.6% [38]. The incidence of HIT is influenced by the underlying disease or indication for heparin therapy. For example, HIT incidence is high in patients with cardiopulmonary bypass (CPB), sepsis, acute hemodialysis (HD), while the incidence is very low in the patients with potential venous thromboembolism (VTE) or chronic HD. Thus, the incidence of HIT for UFH and LMWH must take into account the underlying disease state of the patient. It is only in this context that there is a reasonable indication to prevent/treat VTE.

The differential immunogenicity and incidence of HIT with UFH and LMWH were first noted in a study by Warkentin et al. [39]. In this study of over 700 orthopedic patients randomized to receive prophylactic doses of UFH or LMWH, a lower incidence of HIT was observed (2.7% UFH vs. 0% LMWH; *p* = 0.0018). Subsequent studies and a meta-analysis of approximately 7300 patients have confirmed nearly a 10-fold lower risk of HIT with use of a prophylactic dose LMWH as compared with UFH [38]. The differential effect, however, is not seen at therapeutic doses of UFH and LMWH [40]. In a meta-analysis of studies using these drugs at therapeutic doses, the incidence of clinical HIT appeared to be comparable (UFH 1.5% vs. LMWH 1.2%) [41].

There is one ULMWH, a chemically synthesized heparin pentasaccharide in clinical use, fondaparinux sodium (Arixtra). Fondaparinux has a linear pharmacokinetic profile, and a longer half-life and does not induce immune HIT compared to UFH and LMWH [42]. Fondaparinux is commonly used for the treatment of patients at risk of HIT despite a lack of approval for this indication [43].

### Chemoenzymatically synthesized ultra-low molecular weight heparins and heparin oligosaccharides

Chemoenzymatic synthesis of heparin oligosaccharides includes two major steps; the first step is to generate the backbone using different nucleotide sugar substrates (uridine 5′-diphospho (UDP)-*N*-acetyl-D-glucosamine and UDP-D-glucuronic acid) and glycosyltransferases, while the second step is to modify the backbone using epimerase and different sulfotransferases [8, 44–46]. There has been great progress in the chemoenzymatic synthesis of heparin derivatives, leading to gram scale production in the research laboratory [9, 13, 18]. A detailed characterization of the interaction of PF4 and HIT antibodies with homogeneous synthetic heparin oligosaccharides of 6-, 8-, 10-, and 12-mer and a hypersulfated 12-mer recently was reported [47]. Pure synthetic heparin oligosaccharides display stronger binding affinity to PF4 than animal-derived heparins of corresponding length. In contrast, HIT antibodies bind weakly to complexes formed between PF4 and heparins ≤8-mer than with complexes formed between PF4 and heparins ≥10-mer. The addition of one sulfate group to the heparin 12-mer resulted in substantial changes in its binding characteristics to PF4 [47].

### Recombinant heparin production by metabolic engineering of CHO cells

Cellular production of heparin provides a single process alternative potentially under cGMP control. The Chinese Hamster Ovary (CHO) cell line has been widely used as an industrial line for producing recombinant therapeutic products, and CHO cells produce substantial amounts of heparan sulfate (HS) [6]. In our previous study, *N*-deacetylase/*N*-sulfotransferase (NDST2) and mouse heparan sulfate 3-*O*-sulfotransferase 1 (Hs3st1) genes were successfully transfected sequentially into CHO cells [17, 48–51]. CHO-S cells, expressing Golgi-targeted Hs3st1 can produce recombinant heparin/HS with an increased anticoagulant activity [17, 48, 49, 51]. We expect that through metabolic engineering, it is possible to direct cellular synthesis of heparin/HS to specific compositions with reduced risk of HIT without changing the potency of anticoagulation. For example, it was reported that removal of 2-*O*-sulfate from HS results in significant increases in the IC_50_ for PF4 binding to heparin in competition formats [52], unfortunately, 2-*O*-sulfate is a required component of the ATIII binding site, critical for anticoagulant activity.

### Biosynthetic heparin

We have produced biosynthetic heparin by *E. coli* K5 fermentation (to prepare heparosan) and chemoenzymatic methods [44]. The heparosan was first chemically converted to *N*-sulfo-heparosan and then modified by a three-step enzymatic process to obtain anticoagulant biosynthetic heparin [10, 20]. Using this approach, multigram quantities of chemically and biologically equivalent biosynthetic heparin to that of USP heparin have been demonstrated. Preliminary studies show that biosynthetic heparin showed a similar interaction profile to PF4 as USP heparin [37]. By fine-tuning enzymatic modification, it may be possible to generate biosynthetic heparin with altered structures giving good anticoagulation activity but reduced risk of HIT.

### Regulatory importance of the development of bioequivalent heparins

Every country has their regulatory bodies that oversee the drug approval process, for example, the European Medicines Agency in European Union and the Food and Drug Administration in the United States [53]. Specifically, the U.S. FDA regulates pharmaceutical and biopharmaceutical drug development and commercialization via the Center for Drug Evaluation and Research (CDER) and the Center for Biologics Evaluation and Research (CBER), respectively. In 1987–88, the regulations of biological products were split between CDER and CBER. CDER regulates prescription drugs, over-the-counter drugs (OTCs), generic drugs, and specific biological products, including, recombinant proteins, monoclonal antibodies (mABs), hormones, thrombolytics and immunomodulators. CBER regulates specific biological products, including, allergenics, blood derivatives, blood and blood products, cellular therapies, and vaccines. As part of the review process, the FDA directs the regulatory pathway for innovator pharmaceuticals, generics, biologics, and biosimilars. Although the regulatory pathways may have certain differences, all the regulatory pathways focus on drug safety and efficacy.

Addressing the HIT and immunogenic potential of biosynthetic heparins during the drug development and approval processes is critical. With the growing interest in developing new non-animal sourced heparins, it is vital to develop robust and sensitive analytical tools to address the HIT potential of biosynthetic heparins. Currently, most available HIT analysis is focused on the clinical management of HIT (e.g., clinicopathological diagnosis of HIT). In vitro assessment, rather than clinical evaluation provides the best prediction of the potential for developing HIT. Development of robust and sensitive analytical and diagnosis tools is critical to evaluate the interaction of heparin and PF4, as well immunogenic potential of the heparin/PF4 complexes. Examples of in vitro and analytical tools towards evaluating the biomolecular interaction of heparin and PF4 may include, but are not limited to: (1) binding kinetics studies (e.g., surface plasmon resonance, SPR); (2) heparin-PF4 ULIC complex size (e.g., electron microscopy); and (3) heparin-PF4 complex charge and stoichiometric ratio (e.g., zeta potential). The applications may include comparative studies of heparins from various sources, evaluating lot variability, and process development of bioequivalent heparins, among others.

## Biology of HIT

HIT (Type II) is immune-mediated drug-associated thrombocytopenia [54]. Type II HIT occurs when specific antibodies bind to the heparin-PF4 complex and lead to platelet activation. Platelet activation, accompanied by other cascading reactions, leads to cerebral sinus thrombosis, DVT, lower limb arterial thrombosis, myocardial infarction, pulmonary embolism, skin necrosis, and thrombotic stroke. If untreated, HIT may be life threatening. The current section focuses on (1) PF4 structure, (2) Biological and clinical importance of PF4, (3) heparin and PF4 interaction, (4) current knowledge of HIT antibodies, and (5) pathophysiology of HIT.

### Platelet factor 4 (PF4, CXCL4)

**Fig. 1:**
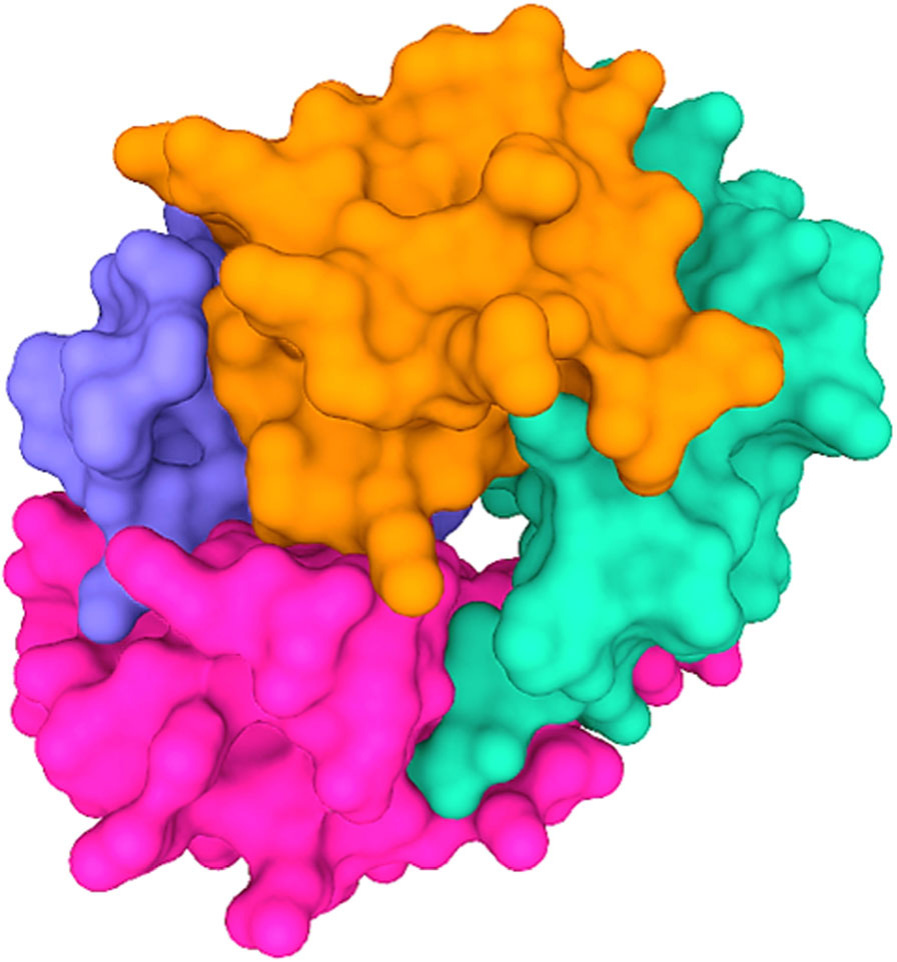
The homotetrameric form of PF4 forms a cylindrical structure and comprises of an equatorial ring. Four PF4 monomers (https://www.uniprot.org/uniprot/P02776, accessed on May 2021) form a homotetrameric complex. The homotetrameric complex bind to highly negative molecules and polyanions, including heparin. PF4 structure and images created using Mol* (D. Sehnal, S. Bittrich, M. Deshpande, R. Svobodová, K. Berka, V. Bazgier, S. Velankar, S.K. Burley, J. Koča, A.S. Rose (2021) Mol* Viewer: modern web app for 3D visualization and analysis of large biomolecular structures. Nucleic Acids Research. doi: 10.1093/nar/gkab314). The structure and information of PF4 (1F1F9Q) was obtained from RCSB PDB (https://www.rcsb.org/structure/1f9q, accessed in May 2021)

Platelet factor 4 (PF4, CXCL4) is a naturally occurring positively charged chemokine. The mature monomeric PF4 protein sequence consists of 70 amino acids (7.8 kDa). At equilibrium, PF4 may exist as a monomer, homodimer, homotrimer or homotetramer. The homotetrameric form of PF4 forms a cylindrical structure and comprises an equatorial ring (Fig. 1). The equatorial ring is composed of positively charged amino acids and forms the binding site for negatively charged polyanions, including heparin. A minimum amount of PF4 must be present in circulating blood in order for anti-heparin PF4 antibody to be formed. Indeed, the presence of PF4 in blood is the most proximal event in the pathogenesis of HIT. However, in recent years, there has been little interest in measuring/studying PF4 prior to the formation of anti-heparin PF4 antibody.

### Biological and clinical importance of platelet factor 4 (PF4, CXCL4)

PF4 may play a role in innate immune responses through binding to polyanionic lipids of bacterial cellular walls (Gram-positive and Gram-negative) [55, 56]. Recently, PF4 also has been associated with vector-based SARS-CoV-2 vaccine-induced thrombosis with thrombocytopenia syndrome (VITT/TTS), thus, emphasizing the clinical significance of PF4 in PF4/polyanion complex formation, and the occurrence of VITT/TTS [57–62]. Administration of ChAdOx1 nCov-19 vaccines results in rare incidents of spontaneous VITT in some patients [63]. Vaccines contaminated with significant amounts of host cell protein (HCP) impurities may be one of the causative factors for VITT [64]. Researchers have demonstrated that ChAdOx1 nCoV-19 (AstraZeneca) contained significant amounts of HCPs, including functionally active proteasomes and adenovirus protein impurities [64]. PF4 can complex with the ChAdOx1 nCoV-19 constituents of this vaccine. Researchers also evaluated the Ad26.COV2.S vaccine (Johnson & Johnson). The Ad26.COV2.S vaccine contained a much lower amount of impurities. Importantly, PF4 did not appear to complex with the purified forms of ChAdOx1 nCoV-19 or with Ad26.COV2.S virions. Researchers showed that ChAdOx1 nCoV-19, but not Ad26.COV2.S, induced vascular hyperpermeability [64].

The role of PF4 in VITT/TTS further reinforces the importance the binding of PF4 with known and unknown polyanions, and the complications that these might pose to patients susceptible to HIT. These findings show the importance of evaluating biosynthetic heparins and their HIT potential. In parallel, these findings also demonstrate how auxiliary factors can complicate the heparin drug development pathway.

### Heparin and PF4 interaction: formation of immunogenic ultra-large complexes

**Fig. 2:**
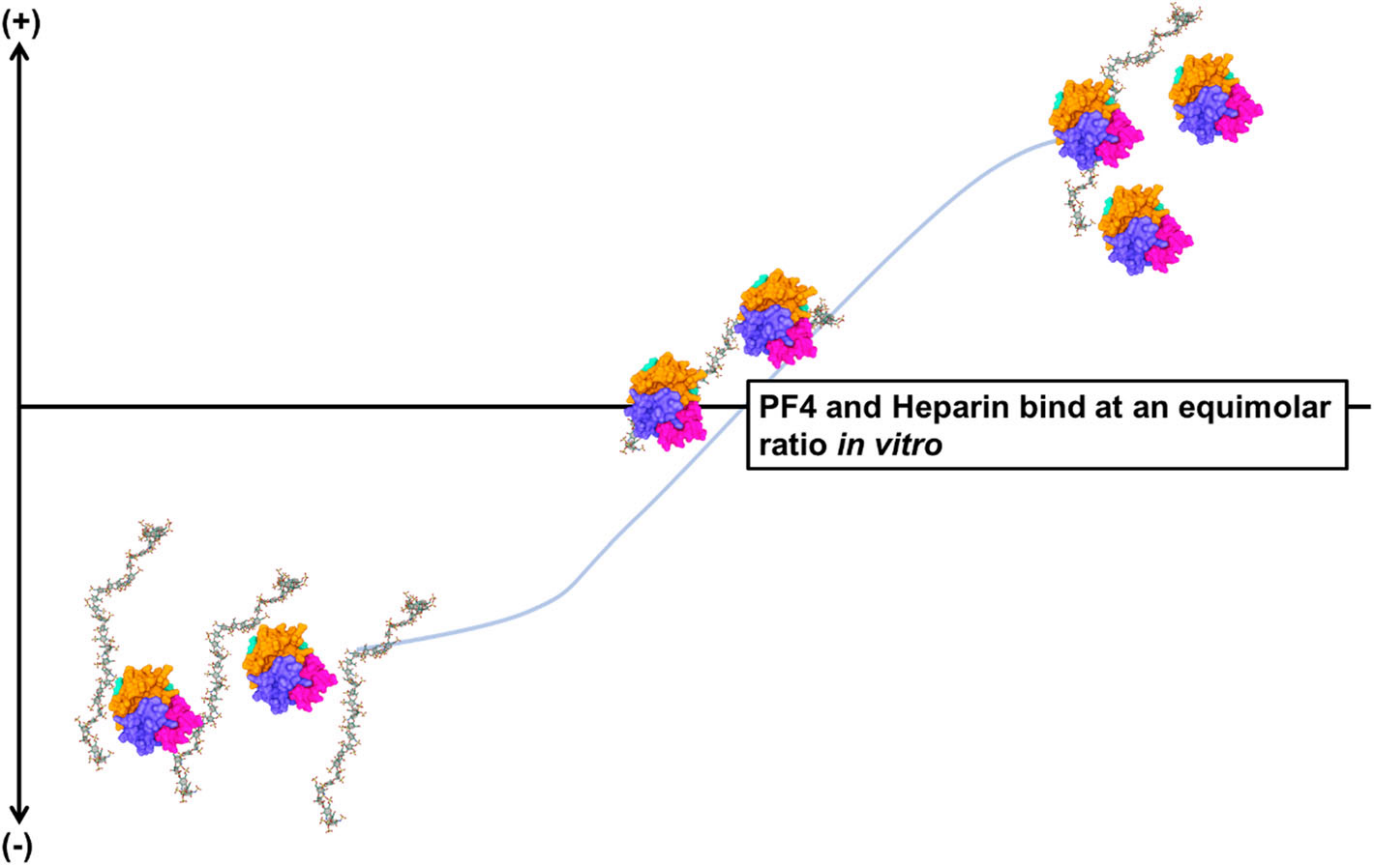
Binding of PF4 and heparin is a stoichiometrically driven phenomenon. Both PF4 and heparin structure and images created using Mol* (D. Sehnal, S. Bittrich, M. Deshpande, R. Svobodová, K. Berka, V. Bazgier, S. Velankar, S.K. Burley, J. Koča, A.S. Rose (2021) Mol* Viewer: modern web app for 3D visualization and analysis of large biomolecular structures. Nucleic Acids Research. doi: 10.1093/nar/gkab314). The structure and information of PF4 (1F1F9Q) was obtained from RCSB PDB (https://www.rcsb.org/structure/1f9q, accessed in May 2021). The structure and information of representative heparin was obtained from RCSB PDB (https://www.rcsb.org/structure/3irl, accessed in May 2021)

The formation of the heparin-PF4 complex is a charge-dependent and stoichiometrically driven phenomenon [23, 52, 65]. Sensitive analytical tools have aided the understanding of biomolecular interaction of heparin and PF4 in vitro, including: (1) PF4-heparin kinetics (e.g., via surface plasmon resonance); (2) PF4-heparin complex size (e.g., via electron microscopy); and (3) heparin-PF4 complex formation (e.g., via zeta potential) [37]. Ultra-large heparin-PF4 immune complexes (ULICs, > 670 kDa) are associated with HIT pathogenesis [23]. The ULICs form at an equimolar ratio of heparin and PF4 in vitro [37] (Fig. 2). The formation of ULICs also depends on the heparin chain size, and heparin chains with at least 12 saccharides are necessary to form ULICs [23]. The incidence of HIT is highest with UFH, fewer with LMWH, and none with fondaparinux [66]. Fondaparinux can bind to PF4 but these complexes are weakly recognized by HIT antibodies. However, it has been reported in certain cases (e.g., aortic stent-graft placement and stroke), patients may develop fondaparinux associated HIT [66–74]. HIT is a complex phenomenon, and with the advances in molecular biology and bioanalytical tools, the role of other factors (e.g., disease status, and/or genetics) in the incidence of HIT may be uncovered.

Patients who have been previously exposed to heparin, and, thus, have been sensitized to the heparin-PF4 immune complex, produce anti–PF4–heparin antibodies [23]. The key anti–PF4–heparin antibodies are immunoglobulin G, (IgG, 80%), and may also include other immunoglobulins, including IgA and IgM [75–78]. Specific antibodies bind to the PF4/heparin complex [79, 80]. In animal models, specific HIT antibodies (e.g., murine monoclonal HIT-like monoclonal antibody) binds to the PF4/heparin complex and elicits HIT [80]. The crystal structure of PF4 interacting with fondaparinux and the HIT-like monoclonal antibody reveals that the PF4 tetramers cluster around heparin, and multiple HIT-like monoclonal antibodies bind to the outer surface of PF4 tetramers [23, 80, 81]. With the development of sophisticated analytical tools, researchers have solved the crystal structures of the: (1) homotetrameric PF4 tetramer complexed with fondaparinux; (2) homotetrameric PF4 complexed with the KKO-Fab (a murine HIT-like monoclonal antibody); and (3) monomeric PF4 complexed with the RTO-Fab (a non-HIT anti-PF4 monoclonal antibody) [82]. KKO (Anti–human PF4 monoclonal antibodies to PF4; KKO causes thrombocytopenia in an in vivo model of HIT) and RTO (Anti–human PF4 monoclonal antibodies to PF4, RTO does not cause thrombocytopenia in an in vivo model of HIT) have been characterized, including their amino acid sequences [83]. This results in the formation of ultra-large HIT immune complexes (ULICs) [23, 80, 81].

ULICs mediate the immune response and subsequently HIT pathogenesis [23]. Certain antibodies (e.g., murine RTO) bind to the PF4/heparin complex but do not form ULICs [23, 80]. Non-pathogenic antibodies bind to PF4 and prevent tetrameric PF4 formation [23, 80]. Tetrameric PF4 is critical for ULIC formation [23]. The researchers further have shown that certain (non-pathogenic) antibodies are HIT blocking antibodies, and inhibit the in vitro and in vivo platelet-activating ability of KKO [23, 80].

### Pathophysiology of HIT

ULIC recognizes and binds to specific cell surface receptors on, platelets, monocytes, neutrophils, and endothelial cells [84]. For example, binding of the HIT immune complex to FcγRIIa (CD32) platelets induces platelet activation, aggregation, and degranulation [85]. Platelet activation leads to the release of procoagulants, microparticles and serotonin. The release of procoagulants leads to cascading events, including thrombin generation and thrombosis. The release of serotonin is a direct measure of heparin-dependent platelet activation [84]. The presence of HIT antibodies in a patients’ serum can be assessed using a serotonin release assay [84]. Platelet aggregation leads to a decrease in platelet count (< 1.5 × 10^11^/L or < 50% of baseline platelet count) [84]. The decrease in platelet count, with the addition of other associated symptoms, may indicate HIT and can be used in HIT clinicopathological diagnosis (discussed below). The binding of ULICs to monocytes leads to tissue factor (TF) expression on the monocytes and peripheral blood mononuclear cells (PBMCs) [23, 85–87]. An increase in TF leads to activation of the blood coagulation cascade and the generation of coagulation proteases, such as Factor Xa (FXa) and thrombin [23, 85–87]. HIT antibodies can also interact with other glycosaminoglycans, including HS present on endothelial cells [23, 85–87]. This interaction can cause activation of TFs and a cascading reaction resulting in endothelial damage. The activation of TFs also may lead to increased production of thrombin and platelet activation [23], the latter also leading to the release of additional PF4 and subsequently aggravating the symptoms of HIT [23].

## Clinicopathological diagnosis of HIT

The clinicopathological diagnosis of HIT includes 4 T scoring system and laboratory testing [84, 88–90]. Various laboratory tests are commonly used to evaluate HIT diagnosis, and fall under two broad categories, immunoassays that evaluate the presence of HIT antibodies (e.g., anti-PF4-heparin enzyme-linked immunoassays) and functional assays that evaluate the ability of the HIT antibodies to activate platelets (e.g., serotonin release assays, SRA). The combinatorial knowledge from 4 T score analysis and laboratory tests may prevent misdiagnosis. Moreover, understanding the principles, advantages, and drawbacks of the 4 T scoring system and laboratory tests may aid in further development of bioequivalent heparins with low propensity of causing HIT.

### 4 T scoring system

The 4 T scoring system is a validated, clinical risk analysis tool and utilizes clinical findings to estimate the probability of HIT [91–95]. Four factors define the 4 T score analysis: (1) the timing of onset of platelet count decrease; (2) thrombocytopenia; (3) thrombosis or other sequelae; and (4) other causes of thrombocytopenia (Table 2). A high 4 T score (> 5 points) and intermediate 4 T score (4–5) is indicative of a high probability of HIT and requires further laboratory testing [96]. A low 4 T score (0–3) may not be indicative of likelihood of HIT [96]. The 4Ts score has a negative predictive value (NPV) of approximately 100%. The limitations of the 4 T scoring system, such a significant inter-observer variability and modest positive predictive value (PPV), have led to exploration of alternate scoring systems [97].

**Table 2:** 4 T Scoring system: A pretest score for evaluating probability of HIT

Factors	2-points	1-point	0-point
**The timing of onset of platelet count decrease**	• Clear onset of platelet count decrease (5–10 days) • Platelet count decrease ≤1 day, if last heparin exposure is within 30 days	• Suspected 5–10 days but was not documented or missing • Onset of platelet decrease after 10 days or ≤ 1 day, with the last heparin exposure at 30–100 days ago	• < 4 days with no recent heparin exposure
**Thrombocytopenia**	• Platelet count fall is > 50%and • Platelet nadir ≥20 × 10^9^ /L	• Platelet count fall is 30–50%or/and • Platelet nadir = 10–19 × 10^9^ /L	• Platelet count fall is < 30% Or/and • Platelet nadir < 10 × 10^9^ /L
**Thrombosis or other sequelae,**	• New thrombosisor/and • Skin lesions or necrosis at the heparin injection sitesor/and • Acute systemic reaction (post-intravenous heparin injection)	• Progressive thrombosisand/or • Recurrent thrombosis Or/and • Non-necrotic (erythematous) skin lesions Or/and • Silent, suspected	• None
**Other causes of thrombocytopenia**	None	Possible	Defined

### Lab testing: immunoassays

Immunoassays can be used to evaluate the binding of HIT antibodies to PF4/heparin (or PF4/polyanion) complexes [91, 95, 98], which may result in immune thrombosis and thrombocytopenia. Rare incidents of spontaneous vaccine-induced immune thrombotic thrombocytopenia have occurred in some patients who were administered with the ChAdOx1 nCov-19 vaccines [63, 99]. The most widely used immunoassays for assessing HIT are PF4-dependant enzyme immunoassays (EIAs) [91, 100, 101]. The principal of the assay is based on enzyme-linked immunosorbent assay (ELISA) [91, 100, 101]. The EIAs are rapid and commercially available, and often exhibit high sensitivity (95–97%) and high negative predictive value [98]. However, EIAs may lack high specificity and positive predictive value [98]. The lack of specificity arises from the fact that EIAs detect total HIT antibodies. Other available immunoassays include the particle immunofiltration assay (PIFA) and functionalized immunoassay (e.g., latex immunoturbidimetric assay, LIA).

### Lab testing: functional assays

Functional assays are used to test whether patient-derived HIT antibodies can induce platelet activation (from healthy donors) in a heparin-dependent manner [89, 92, 95, 101–103]. The tests are performed using whole blood (WB), platelet-rich plasma (PRP), or isolated washed platelets (WP) from healthy donors [89, 92, 95, 101–103]. Serotonin release assay (SRA) is the gold standard for HIT diagnosis [89, 103]. The SRA evaluates the ability of patients HIT antibodies to activate donor platelets. HIT antibodies will activate platelets and release serotonin, which directly correlates to a positive HIT diagnosis [89, 103]. This assay is sensitive to HIT antibodies and is highly specific (> 90%) [89, 103]. However, the SRA is expensive and time-consuming [89, 103].

Other functional assays include those based on platelet aggregation and measurement of specific biomarkers on activated platelets [89, 92, 95, 101–103]. Functional assays based on the principles of platelet aggregation include heparin-induced platelet aggregation (HIPA), light transmission aggregometry (LTA), and heparin-induced multiple electrode aggregometry (HIMEA) [89, 92, 95, 101–104]. Functional assays based on flow cytometry, include analysis of increased expression of CD62P (P selectin) or phosphatidylserine expressions on activated platelets [89, 92, 95, 101–103].

## Conclusions

Heparin is an essential life-saving drug and is extensively used as an anticoagulant. However, HIT Type II is an adverse immune-mediated effect of heparin therapy. HIT occurs when specific antibodies bind to PF4/heparin complexes and result in cascading immune response, leading to thrombosis and thrombocytopenia. HIT is life threatening, although it occurs only in a small fraction of patients undergoing heparin therapy, suggesting a role of other contributing factors, which may include disease status and genetics. Current developments in COVID and vaccine research have also highlighted the clinical significance of PF4 in PF4/polyanion complex formation and incidence of vaccine-mediated thrombocytopenia.

Emerging efforts towards biosynthetic heparins from non-animal sources, have led to an increasing interest in understanding the biology of HIT, and developing safe and effective biosynthetic heparins. Knowledge of the biology of immune-induced thrombosis and thrombocytopenia may aid in predicting HIT potential of bioequivalent heparins.

## Data Availability

Not applicable.
